# The Laser Therapy for Hemorrhoidal Disease: A Prospective Study

**DOI:** 10.7759/cureus.19497

**Published:** 2021-11-12

**Authors:** Rafique Umer Harvitkar, Giri Babu Gattupalli, Seshu Kumar Bylapudi

**Affiliations:** 1 General Surgery, Dr Lakhumal Hiranand Hiranandani Hospital, Mumbai, IND; 2 Surgery, Sri Chandra Sekhara Hospital, Hosur, IND; 3 General Surgery, Pilgrim Hospital, Boston, GBR

**Keywords:** prospectively, laser procedure, acute hemorrhoidal syndrome, rectal prolapse, hemorrhoidal disease

## Abstract

Aim: This prospective study aimed to determine the outcomes and postoperative complications of hemorrhoid disease (HD) treated by hemorrhoidal laser procedure (HeLP).

Background: We, herein report the results of 18 months of methodical use of mini-invasive laser procedures in 100 patients with grades 2 and 3 hemorrhoids and minimum to a mild degree of rectal prolapse. The surgical technique is called HeLP.

Methods: Data were collected on the duration of the procedure, intraoperative complications, postoperative pain, the declivity of hemorrhoids, persistency or complete resolution, and recurrence of hemorrhoids were collected prospectively.

Results: No evidence of intraoperative complications occurred. The median follow-up was nine months. Postoperative pain was not significant or null in most patients. There was no rectal tenesmus or alteration of defecation habits. Plateau of hemorrhoid symptoms and downgrading of hemorrhoid size reached approximately three to seven months post-procedure. The frequency of pain, bleeding, pruritus ani, and acute hemorrhoidal syndrome decreased by 75-80%. There was a significant reduction in hemorrhoids with the rate of recurrence being 7% over 12 months of follow-up.

Conclusion: Our study evaluated and demonstrated that HeLP is an effective, safe, and non-painful procedure for the management of patients with the symptomatic second or third degree of hemorrhoid with mild to the minimum degree of rectal mucosal prolapse. It is a suitable ambulatory treatment.

## Introduction

Hemorrhoids are one of the most common ill effects of urbanization. Among colorectal diseases, hemorrhoidal disease (HD) ranked number one with prevalence ranging from 3% to 29% with more than 4% of the symptomatic population [1]. Male are more frequently affected than females. Hemorrhoids are graded into different grades from 1 to 4. Grade 1 is seen as a congested vein on an anoscopic examination. Grade 2 prolapse but reduces spontaneously. Grade 3 hemorrhoids are prolapsed but require manual reduction whereas grade 4 are irreducible [2]. The most common symptoms are pain, bleeding, Pruritus ani, prolapse, or mucosal discharge. Hemorrhoidal acute syndrome (HAS) can also be part of present symptoms. It was stated as the presence of pain, bleeding, and mucous discharge lasting for more than five days in the last year. There are many methods of HD treatment ranging from conservative, band ligation, sclerotherapy, stapled hemorrhoidopexy, laser photocoagulation to Milligan Morgan (MM). MM is the gold standard and most frequently used procedure. However, postoperative pain, hemorrhage, urinary retention, and abscess formation are the most common side effects associated with MM. The long-term complications include stool incontinence, fistula formation, stenosis [3,4]. Because of these drawbacks, Burch et al. have come up with a new no excisional surgical procedure known as hemorrhoidal laser procedure (HeLP) [5]. It is mini-invasive and mostly suitable for a symptomatic second to third-degree with minimal rectal prolapse. With HeLP, it is possible to get a significant reduction and shrinkage of final branches of the superior hemorrhoidal artery [6,7]. Usually, no anesthesia is necessary for the procedure. In our prospective series, we reported the results of 18 months of methodical use of HeLP in patients with symptomatic second /third-degree HD with mild to moderate rectal prolapsed for 100 Patients.

## Materials and methods

From February 2017 to August 2018, 100 patients underwent the HeLP procedure at our institute. Informed and written consent was taken for all patients considered for the study (reference ID of Ethic: 1103/PUQ/2QA/P30). Preoperatively, all patients underwent thorough medical evaluation, biochemical blood analysis (routine), and physical examinations (digital rectal and/ or proctoscopic examinations). The HeLP was recommended to symptomatic second/third-degree HD with mild to moderate rectal prolapse. The rectal prolapse was assessed on digital and or anoscopic examination. Defecography was limited to those with symptoms of obstructed defecation syndrome. Those patients who fulfill the following criteria were subjected to HeLP in our institute. Inclusion criteria were (i) patients with second/third-degree symptomatic HD with minimum to mild rectal prolapse; (ii) conservative treatment failure. Our exclusion criteria for the HeLP performance were (i) patients younger than 18 or >75 years; (ii) third-degree HD with severe prolapse; (iii) fourth-degree HD; (vi) thrombosed hemorrhoidal cushion, fecal incontinence, obstructed defecation syndrome, anal stenosis anal fissure, anal fistula; (v) acute inflammatory bowel disease; (vi) surgical anastomosis of <5 cm from the dentate line.

Time is taken for the procedure, preoperative complications (bleeding), postoperative pain, HD downgrading, resolution, and relapse were collected via the prospective method. A verbal rating scale (VRS) was used to evaluate 4 points (non, mild, moderate, severe) postoperative pain. No pain was evaluated as zero mild pain=1, moderated=2, severe=3 on the scale. All patients were evaluated 7 days and 28 days post operating then at three months, six months, and nine months post-surgery.

All the surgeries were performed using the HeLP kit. A proctoscope with a specific diameter of 24 mm was introduced into the anal canal (rectum) with the patient in the lithotomy position. Almost all the procedures were performed without general or local anesthesia except for three patients where for one patient we used general anesthesia, and for the remaining two patients, mild sedatives were used to counter the effects of anxiety. No antibiotics were routinely used. No bowel preparation was needed; however, two enemas were given to the patients (the evening before and morning of the procedure).

Through a small window of the proctoscope approximately 4 cm above the dentate, the terminal divisions of the hemorrhoidal (superior) artery were identified with the help of a Doppler probe (transducer). Once the identification was done, all the branches were lasered (close) using laser optic fiber (6 pulses of 1.1 seconds each with 0.5 seconds pause with the energy of 12 W), which reinstate the probe in the same small aperture of a proctoscope. The effects of the laser were seen by reintroducing the doppler probe. If required, another two new sets of laser shots were delivered at the same point. Pain killer administered only If a patient asked. Almost all patients were discharged within a few hours of surgery.

Statistical analysis was followed out with the assistance of the Fisher exact test when appropriate for categorical variables. 94% confidence interval and odds ratio were calculated. The Mann-Whitney-U- test was applied to monitor continuous variables which are not normally distributed 'D'Agostino-Pearson test was used to determine the normality of variable distributions. Less than 0.05 of the P-value was considered statistically significant. Macalc software version 10.02.01 was used to perform statistical analysis.

## Results

The age (median) of the participants signed up in the prospective study was 48 years (male 54 and female 46 years; IQR 35-60 years; range 27 to 72 years). Overall, 52 patients (52%) had grade 2, and 48 patients (48%) had grade 3 hemorrhoids. The most common symptom for any grade was bleeding in 66 (66%) cases, pain in 32 (32%), and itching in 20 (20%) of patients. HAS recorded in 32 (32%) of cases.

One patient was given general anesthesia and two patients received mild sedation during the procedure because of the high level of anxiety. Whereas the rest of the surgeries were performed without anesthesia, only 10 (10%) patients needed post-operative painkillers. The median time for the procedure was 19 minutes (IQR 15 to 25: range 10-38 minutes). A median number of nine (IQR 8-11, range 6-12) arterial branches were identified and managed with laser. There was no evidence of significant intraoperative complications. There was minor intraoperative bleeding in eight (8%) patients. It was successfully managed with a laser. However, in four (4%) patients, hemostatic sutures were taken to control the bleeding with round-bodied mersilk or vicryl. These four patients were kept for overnight observation, however, the remaining 96% of patients were discharged six to eight hours post-surgery. Every individual embroil was able to manage and recapitulate their daily activities almost immediately postoperatively.

The median follow-up was nine months (IQR 09-19; range 3-9 months). On day 1 of surgery (84%) of patients observed 0-1 pain VRS. The remaining 16 (16%) patients had moderate pain and they were given pain killers (Diclofenac sodium/Paracetamol) an oral option for pain relief for 24-48 hours. Median pain at post one week of surgery was 0 (IQR 0-1; range 0-1) on the VRS scale. Approximately, 81 (81%) of cases post the first week of HeLP showed fibrin deposition covering all sites of laser penetration sites, whereas 19 patients (19%) had only scanty evidence of the laser procedure been performed. Four weeks after the HeLP no scar or signs of complications were observed on proctoscopic examination. Median pain at four weeks post-surgery was 0 (IQR 0 to 1 range 0-1) on VRS, however, moderate pain was observed in 12 (12%) patients. It was associated with defecation in five (5%) patients, after defecation in four (4%) and pain unrelated to defecation was seen in three (3%) individuals. No relationship was seen between pain severity with preoperative grades of hemorrhoids (7 of 12 patients had grade 3 HD; P=0.36). Eighteen (18%) patients reported mild bleeding analogous to steady HD (68% recovery). The itching was observed in 07(7%) of patients post four weeks of follow-up. The inflated significance (odds ratio 14; 94% interval for confidence three to 51: P=0003) results were noted in this series. None of the patient's complaints of rectal tenesmus or defection habit changes. One month postoperatively (HeLP) 75 (75%) of cases showed downgrading of hemorrhoids; 42 of 52 (80%) patients with preoperative grade 3 HD improved to grade 2 and 40 of 48 (83%) improved from grade 2 to grade 1 of HD.

The improvement was not significantly related to preoperative HD grades (P=0.75). It has been observed furthermore that 4 of 52 (8%) cases with grade 2 HD and 6 of 48 (13%) with grade 3 HD preoperatively; the symptomatic improvement was related to the size of hemorrhoids reduction (P=0.5). The overall symptomatic improvement was in 87% of cases. The symptomatic improvement and grade downgrading for HD is shown in Table 1. Drastic improvement in symptoms and HD downgrading was observed for three months. At this observation, the frequency of pain, hemorrhage, and pruritis reduced by 74.8%, 79%, and 72%, respectively. There was evidence of HAS decreased by 77%. The hemorrhoidal disease grade showed a significant reduction (up to grade 1) in 87% of individuals. The recurrence of HD was 6% at nine months. Two patients had mild symptoms of fecal incontinence at four weeks of follow-up. They were advised pelvic floor exercise, dietary changes. On three months of follow-up, the symptoms of incontinence were resolved. No major complications were recorded at long-term follow up specifically fistula or anal canal stenosis.

**Table 1:** Patients demographics and post HeLP follow up HD: hemorrhoid disease, HAS: hemorrhoidal acute syndrome, HeLP: hemorrhoidal laser procedure

Characteristics	Results	Follow-up post one month of procedure % (n)	Follow-up post three months of procedure % (n)	Follow-up post six months of procedure % (n)	Follow-up post nine months of procedure % (n)
Age (median)	48 years				
Sex: male	54 (54%)				
Female	46 (46%)				
Average time per procedure	19 minutes				
Hemorrhoidal branches identified	8-9 branches				
Symptoms					
Pain	32 (32%)	12 (12%)	9 (9%)	5 (5%)	2 (2%)
Bleeding	66 (66%)	18 (18%)	8 (8%)	6 (6%)	4 (4%)
Pruritis ani	20 (20%)	07 (7%)	4 (4%)	2 (2%)	1 (1%)
HAS	32 (32%	09 (9%)	8 (8%)	5 (5%)	2 (2%)
HD grade					
First	0	42 (42%)	44 (44%)	36 (36%)	10 (10%)
Second	52 (52%)	46 (46%)	45 (45%)	30 (30%)	8 (8%)
Third	48 (48%)	12 (12%)	11 (11%)	6 (6%)	3 (3%)
Complications post-procedure					
Fistula		None	None	None	None
Anal stenosis		None	None	None	None

## Discussion

The hemorrhoidal artery ligation (HAL) via the doppler route is a relatively modern procedure. The Kazumasa Morinaga (Japanese) in 1995 first delineate this technique. With the help of the Doppler technique, they spotted the divisions of hemorrhoidal arteries. Maria et al. validated in their research that HAL is a secure and practically potent substitute procedure to conventional hemorrhoidectomy [8-10]. However, Pucher et al. proposed a non-excisional mini-invasive surgical method called HeLP. The goal of the procedure is the same as HAL where the laser is being used to shrink the terminal branches of HA [11-13].

In our prospective study, the HeLP procedure showed remarkably good results in terms of HD-related symptoms and recurrences. These results validate the vascular theory in the pathophysiology of HD. It is based on the fact of non-capillary interposition of arteriovenous hemorrhoidal shunting system. It also substantiates the dilatation of hemorrhoidal venous plexus which is due to arterial swarming in the superior HA [10,12,14]. Due to lack of vascular connection and flow to pile mass, there is a significant reduction of pile mass and betterment of symptoms.

Laser coagulation of vessels has the advantage of conservation of anatomy and physiology of anal canal, compared to other forms of treatment. Thus, it minimizes post-operative impaired anal function [15,16]. Due to the highly selective effects of laser beams effect on arteries, the damage caused to the surrounding area is minimum. Significantly no or minimum scar tissue with less retraction of rectal mucosa. Given less mucosal retraction, one should observe strict inclusion criteria. Such treatment should be reserved for those with second/third-degree HD with minimum to moderate rectal mucosal prolapse [17,18]. Despite these restrictions, we observed interesting results. Our study revealed that postoperative pain was graded 0-1 on VRS. There was a marked improvement in preoperative symptoms in more than 78% of patients, and a downgrading of the HD in more than 85-87% of the patients within one to three months of the procedure. The patient with HD grade modification experienced symptomatic relief other than those who presented with HD reduction and described a betterment of symptoms presented preoperatively.

Our results confirmed the good finding previously done and reported by other authors; however, it would be useful to learn the rate of symptom recurrence and downgrading of HD on a perpetual basis. It is also important to note that the laser procedure is more expensive than the others, but the cost of the procedure can be cut down by reduced duration of the procedure, no anesthesia, and shorter hospital stay.

## Conclusions

It is often encountered that the severity of hemorrhoids and their size are not correlated well. Severe-looking hemorrhoids with third- to fourth-degree prolapse can cause mild symptoms in some patients. When there is an initial prolapse in second-to third-grade hemorrhoids, the patient may experience itching, bleeding, and recurrent acute pain. In such cases, excisional techniques or stapled hemorrhoids are commonly overtreated. In such cases, the HeLP procedure may be considered a more suitable choice when the conservative mode of treatment fails. Our results demonstrated that Help could be a secure, compelling, and effortless technique for the treatment of symptomatic second to third-degree HD with negligible or minimum prolapse of rectal mucosa. Besides, this technique is also minimally invasive, does not require anesthesia, can be performed as an outpatient procedure, has the advantage of short hospital stay, and faster recuperation.
